# Electrochemical Sensor for Rapid and Sensitive Detection of Tryptophan by a Cu_2_O Nanoparticles-Coated Reduced Graphene Oxide Nanocomposite

**DOI:** 10.3390/biom9050176

**Published:** 2019-05-08

**Authors:** Quanguo He, Yaling Tian, Yiyong Wu, Jun Liu, Guangli Li, Peihong Deng, Dongchu Chen

**Affiliations:** 1 School of Life Sciences and Chemistry, Hunan University of Technology, Zhuzhou 412007, China; hequanguo@126.com (Q.H.); tianyaling0212@163.com (Y.T.); wyy5082010@163.com (Y.W.); liu.jun.1015@163.com (J.L.); guangli010@hut.edu.cn (G.L.); 2 Key Laboratory of Functional Metal–Organic Compounds of Hunan Province, Key Laboratory of Functional Organometallic Materials, University of Hunan Province, Department of Chemistry and Material Science, Hengyang Normal University, Hengyang 421008, China; 3 School of Materials Science and Energy Engineering, Foshan University, Foshan 528000, China

**Keywords:** cuprous oxide, electrochemical reduced graphene oxide, tryptophan, voltammetric detection

## Abstract

In this paper, a nanocomposite of cuprous oxide and electrochemically reduced graphene oxide (Cu_2_O–ERGO) was prepared by a simple and low-cost method; hereby, a new method for the electrochemical determination of tryptophan (Trp) by this composite modified glassy carbon electrode (GCE) is proposed. The prepared materials and modified electrodes were characterized by scanning electron microscopy (SEM), X-ray diffraction (XRD), and cyclic voltammetry (CV). The results showed that Cu_2_O–ERGO/GCE had good electrocatalytic activity for Trp. The effects of supporting electrolyte, scanning rate, accumulation potential, and accumulation time on the determination of Trp were studied. Under the optimum experimental conditions, Trp was quantitatively analyzed by square-wave voltammetry (SWV). The oxidation peak current of Trp had a good linear relationship with its concentration in the range of 0.02–20 μM, and the detection limit was 0.01 μM (S/N = 3). In addition, the modified electrode has high sensitivity, good repeatability, and long-term stability. Finally, the proposed method has been successfully applied in the determination of Trp concentration in practical samples.

## 1. Introduction

It is well known that amino acids play an important role in neuroregulation, organ functioning, and metabolism in human beings and animals. Tryptophan (Trp) is an important amino acid that is a precursor of neurotransmitter serotonin and the neurohormone melatonin. It is also essential to the establishment and maintenance of a positive nitrogen balance in human nutrition [1]. Unfortunately, there is an inadequate amount of Trp in vegetables and fruits, and humans and animals cannot synthesize Trp themselves, so Trp must be obtained from food and pharmaceutical formulas. The World Health Organization recommends a Trp intake of 4 mg/kg per day. However, when Trp metabolism malfunctions, toxic waste is produced in the brain, leading to hallucinations and illusions and resulting in neurological dysfunction [2,3]. Therefore, it is important and urgent to establish a simple, sensitive, and selective method for the determination of Trp in food, pharmaceuticals, and living bodies.

So far, there have been many analytical methods for Trp determination, such as high-performance liquid chromatography (HPLC) [4,5], fluorescence [6], capillary electrophoresis [7], chemiluminescence [8], and spectrometry [9]. Although the above methods are useful for the quantitative analysis of Trp, most have the disadvantages of high cost, complicated sample pretreatment process, long response time, and the requirement for human-sized instruments. Electrochemical methods are good choices to solve these defects because of their high accuracy, high sensitivity, simple operation, and low cost. However, the electron transfer process is slow and the overpotential is high for the direct oxidation of Trp on bare electrodes [2]. In addition, some electroactive biomolecules often coexist with Trp in body fluids, which may have a similar oxidation peak potential with Trp and interfere with Trp determination. Chemical modification of electrodes with suitable materials could solve these problems. Therefore, many efforts have been made in recent years to find materials suitable for the design and construction of high-performance electrochemical sensors to reduce the overpotential and improve the sensitivity [10,11,12,13,14,15,16,17,18,19,20]. However, as shown in Table 1, most of them have deficiencies, such as narrow linear range, insufficient low detection limit, or poor stability. The development of enhanced materials and novel sensors for Trp is still of great significance.

Recently, metal oxide nanoparticles have attracted attention in industry and the scientific research field due to their advantages such as high catalysis, low cost, and good stability [21,22,23,24,25,26,27,28]. In recent years, Cu_2_O nanoparticles, important p-type semiconductors, have been widely used in solar energy conversion, catalysis, lithium-ion batteries, optical devices, and gas sensors [29,30,31]. Cu_2_O is considered a good additive for composite materials, not only because of its low cost and remarkable catalytic activity, but also because of its good chemical stability, low toxicity, abundance, and easy preparation and functionalization. Up to now, Cu_2_O nanoparticles with different morphologies have been synthesized, including nanowire [32], nanooctahedron [33], nanocube [34], and nanosphere [35]. The results show that the structural flexibility of Cu_2_O improves its intrinsic electronic and catalytic properties. Graphene, a carbon-based material, is becoming more and more popular as a nanomaterial in electrochemistry due to its unique electronic, chemical, and structural characteristics. Graphene exhibits many excellent properties including high surface area, high conductivity, and low intrinsic size, which makes it attractive as a heterogeneous catalytic matrix [36,37,38]. Based on these advantages, it has been reported that graphene combined with Cu_2_O can be used for electrocatalytic oxidation of dopamine [39,40], hydrogen peroxide [41,42], and glucose [42,43,44]. These examples confirm that graphene–Cu_2_O nanostructures exhibit better catalytic performance in electrochemistry than single components. The synergistic effect of graphene–Cu_2_O may come from the following aspects: (i) electronic coupling between Cu_2_O and graphene can greatly improve the catalytic activity; (ii) the conductivity of graphene–Cu_2_O composites is higher than when using Cu_2_O alone; (iii) Cu_2_O can reduce the aggregation of graphene nanosheets and improve the chemical stability with the utilization of catalysts. The incorporation of Cu_2_O particles into graphene can improve the electrochemical response of Trp. Therefore, we hope to develop a high-performance electrochemical sensor for Trp.

It is well known that graphene is generally obtained by the chemical reduction of graphene oxide (GO) with hydrazine hydrate or sodium borohydride. However, graphene is hydrophobic. Because of Van der Waals interactions and strong π–π stacking, it often forms irreversible aggregations, which seriously limits its application in electrode modification [16]. Recently the electrochemical reduction of graphene oxide (GO) to produce graphene has been proposed with different electrochemical techniques such as cyclic voltammetry [45,46] or controlled potential method [47,48], which is time-saving, non-toxic, simpler, and greener than chemical reduction methods. Graphene oxide (GO) is a derivative of graphene that has a large specific surface area and many hydrophilic oxygen-containing functional groups on its hydrophobic surface. Such structural characteristics make it a special surfactant and a good carrier to enhance the dispersion of Cu_2_O in water. Therefore, Cu_2_O nanospheres were prepared using a simple precipitation method and then loaded onto the GO layer. Because the conductivity of GO is poor, the Cu_2_O–GO composites were coated on the surface of a glassy carbon electrode (GCE) and reduced by an electrochemical method. The conductivity of electrochemically reduced GO (ERGO) is much higher than that of GO due to the restoration of conductive carbon conjugate networks. It was found that the prepared material had good electrocatalytic activity for Trp. A detailed study was done on the electrochemical behavior of Trp on the Cu_2_O–ERGO/GCE. Consequently, a simple, efficient, and low-cost electrochemical method for the rapid and sensitive detection of Trp was proposed.

## 2. Experimental

### 2.1. Materials

Cupric sulfate (CuSO_4_·5H_2_O), tryptophan (Trp), graphite powder, hydrazine hydrate solution (80 wt %), polyvinyl pyrrolidone (PVP) and all other reagents were of analytical grade from Shanghai Sino pharm Chemical Reagent Co. Ltd., Shanghai, China. Compound amino acid injections (17AA-I) and (18AA-I) were obtained from Xuzhou the Fifth Pharmaceutical Corporation, Xuzhou, China and Guangzhou Green Cross Pharmaceutical Corporation, Guangzhou, China, respectively. Buffer and standard solutions were prepared using double distilled water. 1.0 mM Trp solution was prepared by dissolving 0.0204 g Trp in a small volume of 1.0 M NaOH solution, and then diluted with water in a 100-mL volumetric flask. Serial dilutions with doubly distilled water were used to prepare more dilute solutions.

### 2.2. Apparatus

The electrochemical measurements were performed with a CHI 660E Electrochemical Workstation (Chenhua Instrument Co. Ltd., Shanghai, China). A conventional three-electrode cell was used at room temperature. A Hg/Hg_2_Cl_2_ (KCl saturated) electrode, a platinum wire electrode, and a bare or modified GCE were used as the reference, auxiliary, and working electrode, respectively. pH measurements were done using a digital pHs-3c Model pH meter (Shanghai Leichi Instrument Factory, Shanghai, China). The morphologies of the nanomaterials were observed by a scanning electron microscope (EVO10, ZEISS, Jena, Germany). The crystal structure of Cu_2_O was collected with a powder X-ray diffractometer (PANalytical, Holland, The Netherlands) with Cu Kα radiation (0.1542 nm). 

### 2.3. Preparation of Cu_2_O and Cu_2_O–GO Composites

Cuprous oxide nanoparticles (Cu_2_O NPs) were synthesized according to a previous report with some modification [39]. Typically, 100 mg CuSO_4_·5H_2_O and 50 mg PVP were dissolved in 20 mL H_2_O under stirring. Then 4 mL of 0.2 M NaOH was added dropwise, and blue precipitate was observed. Finally, 15 μL hydrazine hydrate solution (80 wt %) was added to the above mixture and stirred for 20 min at room temperature and a brick-red suspension resulted. The solid product was separated from the solution by centrifugation, washed with absolute ethanol and water, and dried in vacuum at 60 °C. Graphene oxide (GO) was synthesized referring to our preceding report [16]. Eventually, 1.0 mg of Cu_2_O NPs was mixed with 20 mL of GO solution (1 mg/mL), followed by ultrasonication for 2 h to obtain a homogeneous Cu_2_O–GO dispersion.

### 2.4. Preparation of Modified Electrode

To fabricate modified electrode, bare GCE was polished to a mirror-like state by 0.05 μm α-Al_2_O_3_ slurry. After successive sonication in ethanol and doubly distilled water, the electrode was allowed to dry at room temperature. Typically, 5 μL Cu_2_O–GO nanocomposite was dropped onto the surface of the cleaned GCE with a micro-injector. The obtained electrode was noted as Cu_2_O–GO/GCE. The Cu_2_O–GO/GCE was immersed in a 0.1 M phosphate buffer (pH 6.0) and then electrochemically reduced at –1.2 V for 120 s to obtain Cu_2_O–ERGO/GCE. In a similar way, GO/GCE, Cu_2_O/GCE, and ERGO/GCE were obtained for comparison. When not in use, these modified electrodes were maintained at room temperature.

### 2.5. Electrochemical Measurements

The electrochemical measurements were performed in a traditional electrochemical cell, which contained a Trp standard solution of known concentration and 0.05 M H_2_SO_4_ as supporting electrolyte. Accumulation was carried out at −0.1 V for 120 s under continuous stirring, and then kept for 5 s. In the potential range of 0.50–1.1 V, square wave voltammograms were recorded at a scan rate of 0.1 V s^−1^. After each measurement, the potential scan in the same range was repeated successively 3–5 times in 0.1 M H_2_SO_4_ solution to refresh the electrode surface.

## 3. Results and Discussion

### 3.1. XRD and SEM Characterizations

**Figure 1:**
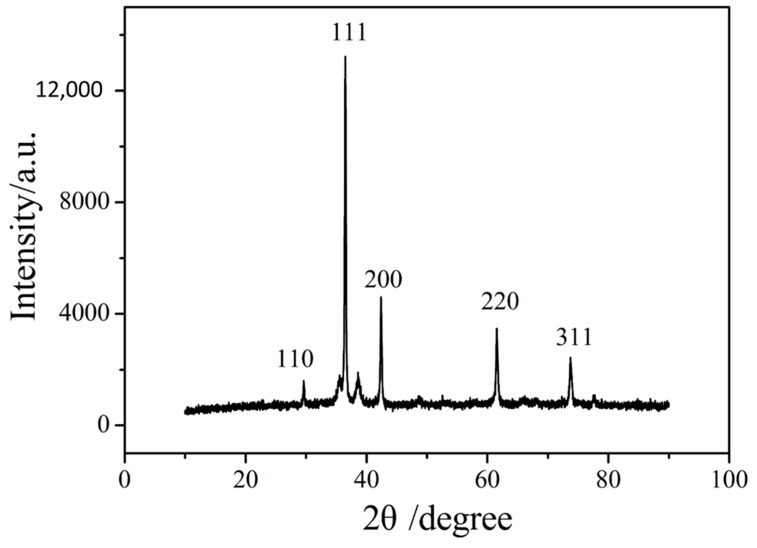
The XRD pattern of Cu_2_O nanoparticles.

Figure 1 shows the X-ray diffraction (XRD) patterns of Cu_2_O nanoparticles. Diffraction peaks are observed at 2θ = 29.6°, 36.5°,42.4°, 61.5°, and 73.7°, which are related to (110), (111), (200), (220), and (311) reflections, respectively. The peak positions are consistent with the standard document of Cu_2_O (JCPDS No. 05-0667) [49], indicating that the prepared material is crystalline Cu_2_O. No diffraction peaks of other possible impurities (such as Cu and CuO) are detected, demonstrating that the product was pure Cu_2_O.

**Figure 2:**
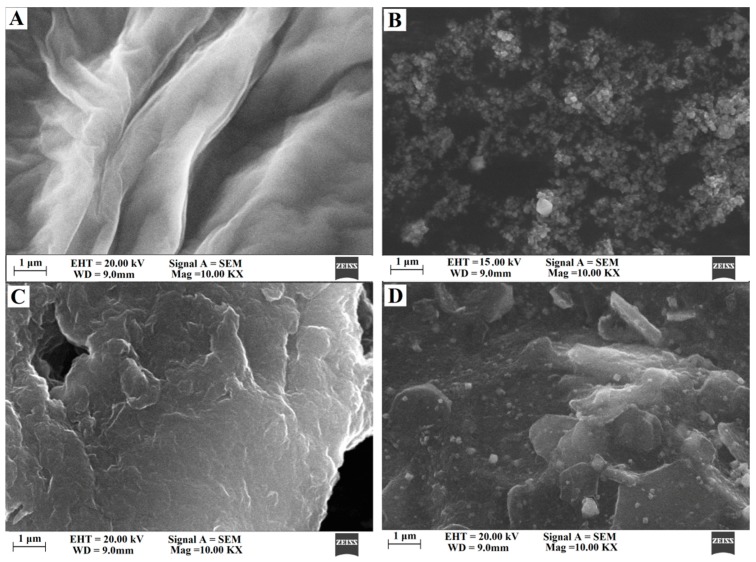
SEM images of (**A**) GO, (**B**) Cu_2_O nanoparticles, (**C**) ERGO, and (**D**) Cu_2_O–ERGO composites.

The surface morphologies of GO, Cu_2_O nanoparticles, ERGO, and Cu_2_O–ERGO composites were characterized by a scanning electron microscope (SEM). As shown in Figure 2A, obvious wavy structures could be observed in the SEM image of GO. Figure 2B shows the SEM image of Cu_2_O nanoparticles. It was found that Cu_2_O nanoparticles are aggregated together, mostly in spherical shape, with an average diameter of about 50–100 nm. As shown in Figure 2C, a lamellar structure of ERGO nanosheets is observed. The layered structure of ERGO can effectively augment the specific surface area of the modified electrode. It can be seen from Figure 2D that a large number of Cu_2_O nanoparticles are evenly distributed on the ERGO with lamellar structure, which indicates that Cu_2_O nanoparticles are well bound to the ERGO carrier. In addition, the SEM images in Figure 2D show that the morphologies and sizes of Cu_2_O nanoparticles are similar to those observed in Figure 2B, showing that the introduction of ERGO does not change the structure of Cu_2_O. 

### 3.2. Electrochemical Characterization of Different Electrodes

**Figure 3:**
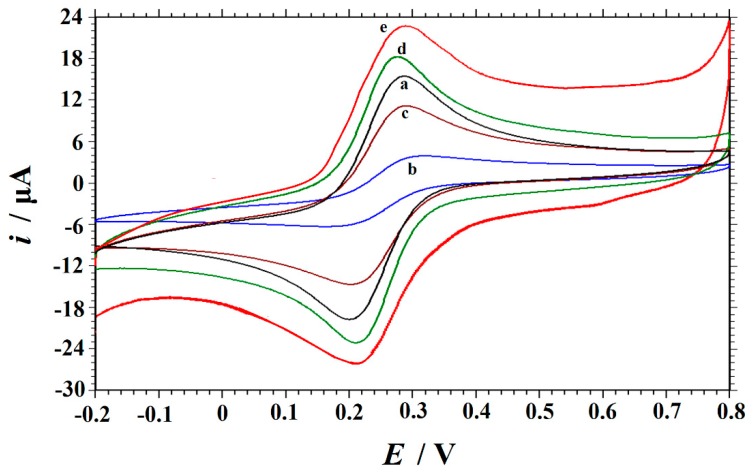
Cyclic voltammograms obtained on (a) GCE, (b) GO/GCE, (c) Cu_2_O–GO/GCE, (d) ERGO/GCE and (e) Cu_2_O–ERGO/GCE in a solution of 1.0 mM K_3_[Fe(CN)_6_] and 0.5 M KCl at the scan rate of 0.1 V s^−1^.

Figure 3 shows the cyclic voltammograms of 1 mM K_3_[Fe(CN)_6_] containing 0.5 M KCl at different electrodes in the potential range of –0.2 to 0.8 V. On the GCE (curve a), the peak to peak potential separation (ΔE_p_) was 87 mV (vs. SCE) with small redox peak currents, corresponding to a quasi-reversible electron transfer process. On the GO/GCE (curve b), the redox peak current of [Fe(CN)_6_]^3−/4−^ declines greatly with ΔE_p_ of 158 mV, which could be due to the weak conductivity of GO. Curve c is the cyclic voltammogram of K_3_[Fe(CN)_6_] obtained at Cu_2_O–GO/GCE with E_pc_ of 0.204 V, E_pa_ of 0.286 V, and ΔE_p_ of 82 mV. As compared with GO/GCE, the peak currents increase and the peak-to-peak separation decreases obviously. The results indicate that Cu_2_O could increase the electron transfer rate of [Fe(CN)_6_] ^3−/4−^ due to its excellent catalytic activity. On the ERGO/GCE (curve d), a pair of well-defined redox peaks also appear with the redox peak current increasing and the ΔE_p_ obviously decreases to 65 mV, which indicates the presence of high conductivity of ERGO. On the Cu_2_O–ERGO/GCE (curve c), the value of ΔE_p_ is 71 mV with the further increase of the redox peak currents, indicating a more reversible electron transfer process. So, the presence of high conductivity of ERGO together with good catalytic activity of Cu_2_O on the GCE surface can further promote the electron transfer and improve the performance of the sensor.

### 3.3. Voltammetric Behavior of Trp at Different Electrodes

**Figure 4:**
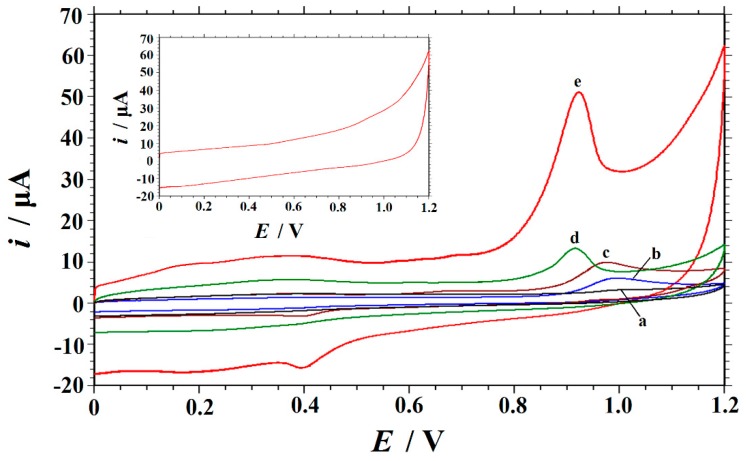
Cyclic voltammograms of 10 μM Trp obtained at different electrodes in 0.05 M H_2_SO_4_ solution. Curve a: bare GCE; curve b: GO/GCE; curve c: Cu_2_O–GO/GCE; curve d: ERGO/GCE; and curve e: Cu_2_O–ERGO/GCE. Scan rate: 0.1 V·s^−1^. Inset: the cyclic voltammogram of Cu_2_O–ERGO/GCE in 0.05 M H_2_SO_4_ solution.

Figure 4 depicts the CV responses of 10 μM Trp in 0.05 M H_2_SO_4_ solution obtained at different electrodes in a potential range of 0.0 to 1.2 V. As can be seen (inset in Figure 4), no redox peaks are observed at Cu_2_O–ERGO/GCE in a blank solution, indicating that the Cu_2_O–ERGO/GCE is non-electroactive in the selected potential region. On the other hand, when 10 μM Trp was added into the blank solution, a well-defined oxidation peak is obtained at +0.923 V in the anodic scan, and a small reduction peak is observed at +0.391 V in the reverse (cathodic) scan (curve d in Figure 4), which could be attributed to the reduction of the oxidized intermediate of Trp [11]. Table 2 lists the peak potentials and currents of Trp obtained at different electrodes. It is observed that the peak current (*i*_p_) of Trp at ERGO/GCE is about 36 times, three times, and two times higher than that of bare GCE, GO/GCE, and Cu_2_O–GO/GCE respectively, which may be attributed to the high conductivity, large surface area, and good catalytic activity of ERGO. The Cu_2_O–ERGO/GCE shows the largest oxidation peak current compared to other electrodes, which are about 120 times higher than that of the bare GCE and almost three times higher than that of ERGO/GCE. The electrochemical data in Table 2 demonstrate that the order of the ability to accelerate the electron transfer is Cu_2_O–ERGO/GCE > ERGO/GCE > Cu_2_O–GO/GCE > GO/GCE > bare GCE. The above results suggest that the Cu_2_O–ERGO/GCE combines the excellent properties of Cu_2_O and ERGO. Specifically, ERGO has excellent electrical conductivity and compensates for the deficiency of semiconductor Cu_2_O. In addition, ERGO provides a larger specific surface area, which increases the loading of Trp, and Cu_2_O immediately exchanges electrons from the Trp loaded in ERGO, promoting the electrocatalytic reaction. Therefore, the nanocomposites can maximize the utilization within a limited electrode surface area, providing an electron transfer microenvironment for accelerating the electrode reaction of Trp and enabling the sensitive determination of Trp. 

### 3.4. Effect of Scan Rate

**Figure 5:**
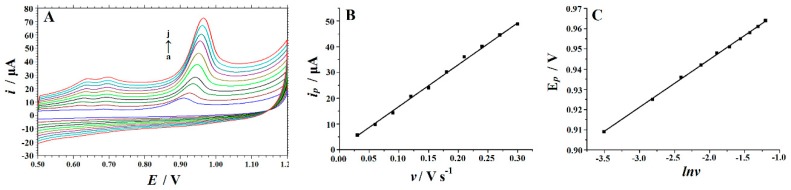
(**A**) Cyclic voltammograms of 10 μM Trp in 0.05 M H_2_SO_4_ solution obtained on the Cu_2_O–ERGO/GCE at different scan rates (a–j: 30, 60, 90, 120, 150, 180, 210, 240, 270, and 300 mV·s^−1^). (**B**) A plot of the peak current versus scan rate; (**C**) a plot of the peak potential versus the Napierian logarithm of scan rate.

The electrode reaction kinetics was studied by investigating the effect of scan rate on the peak current of Trp. Figure 5A indicates the cyclic voltammograms of 10 μM Trp at different scan rates on the Cu_2_O–ERGO/GCE. It can be observed that as the scan rate increases from 30 to 300 mV·s^−1^, the peak current increases gradually and shows a linear relationship with the scan rate. The linear regression equation can be expressed as *i*_p_ = 162.47 *v* + 0.5329 (*i*_p_: μA, *v*: V s^−1^); the correlation coefficient is *R*^2^ = 0.9964, which indicates that the oxidation process of Trp at Cu_2_O–ERGO/GCE is an adsorption-controlled process. The adsorption-controlled behavior was also confirmed by plotting log*i* vs. log*v* corresponding to the equation log*i* = 0.964 log*v*+2.1934 (*R*^2^ = 0.9961). The resulting slope of 0.964 is close to 1.0, confirming the adsorption-controlled properties of the electrode process. As shown in Figure 5C, the peak potential is linearly proportional to the Napierian logarithm of scan rate (ln *v*) in the range of 30–300 mV·s^−1^, the linear regression equation is *E*_p_ = 0.0237 ln*v* + 0.9922 (*E*_p_: V, *v*: V s^−1^), with a correlation coefficient of *R*^2^ = 0.9992. Based on Laviron’s theory [50], the slope of the line is equal to RT/αnF, so αn = 1.08. It is generally believed that α is 0.5 in the completely irreversible electrode process [51], so the number of electron transfer (*n*) involved in the Trp oxidation process is about 2. The results show that at Cu_2_O–ERGO/GCE two electrons were involved in the oxidation process of Trp. 

### 3.5. Optimization of Parameters for Trp Determination

**Figure 6:**
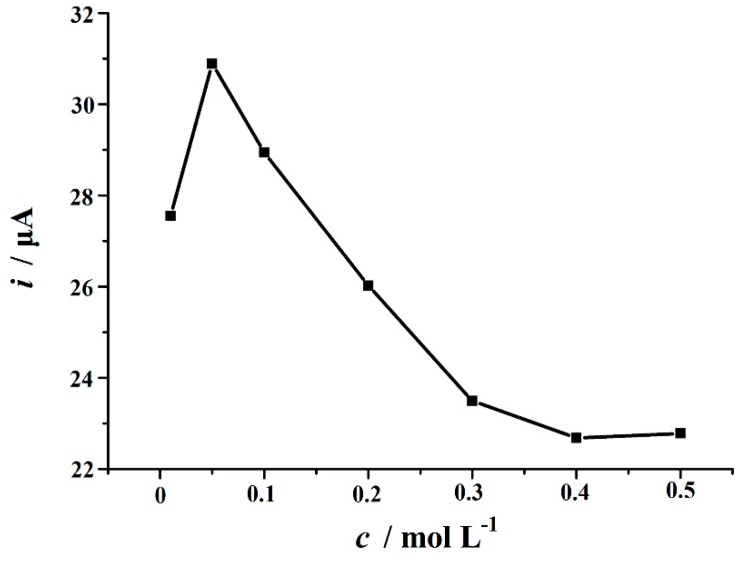
Effect of H_2_SO_4_ concentration on the oxidation of 10 µM Trp on the Cu_2_O–ERGO/GCE.

The effects of voltammetric determination parameters on the electrochemical oxidation of Trp were investigated in detail. First, the effect of supporting electrolyte was evaluated by performing the experiments at different supporting electrolyte including Britton–Robinson buffer (pH 3.0–8.0), phosphate buffer (pH 3.0–8.0), HAc–NaAc buffer(pH 3.0-6.0), (CH_2_)_6_N_4_–HCl buffer (pH 3.0–6.0), HAc–NH_4_Ac buffer (pH 3.0–6.0), NH_3_–NH_4_Cl (pH 8.0–10.0) and some different acids and alkalis such as H_2_SO_4_, HCl, HNO_3_, and NaOH (each 0.1 M). The oxidation peak current values of Trp reached a maximum in H_2_SO_4_ solution. In addition, the effect of the concentration of H_2_SO_4_ solution on Trp oxidation was also evaluated in the range from 0.01 M to 0.5 M. It was found that the oxidation peak current of Trp increased with increasing concentration from 0.01 M to 0.05 M, then gradually decreased from 0.05 to 0.5 M (Figure 6). Therefore, 0.05 M H_2_SO_4_ was used in the current study.

**Figure 7:**
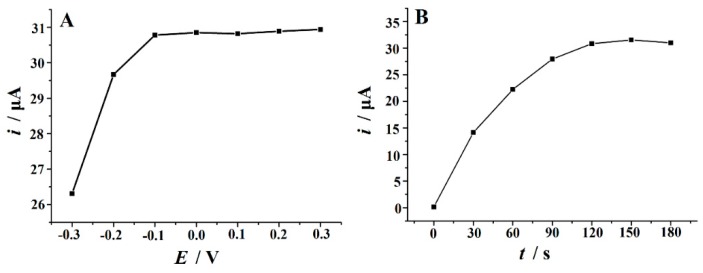
(**A**) The effect of accumulation potential and (**B**) accumulation time on the peak current of 10 µM Trp in 0.05 M H_2_SO_4_ solution on the Cu_2_O–ERGO/GCE at a scan rate of 100 mV s^−1^.

The effect of accumulation potential on the peak current was studied in a 10 µM Trp solution at 120 s accumulation time. It could be seen that, as the accumulated potential increased from –0.3 V to –0.1 V, the peak current increased, and then the peak current changed a little with the further positive shift of the accumulation potential (Figure 7A). Therefore, –0.1 V was chosen as the optimal accumulation potential. The effect of accumulation time on the oxidation peak current of Trp was also investigated at –0.1 V. It was found that with the increase of accumulation time, the peak current of Trp increased gradually. However, beyond 120 s, time period did not lead to significant increment (Figure 7B). Therefore, taking into account the sensitivity together with the analysis speed, 120 s is selected as the optimum accumulation time.

### 3.6. Interference Studies

**Figure 8:**
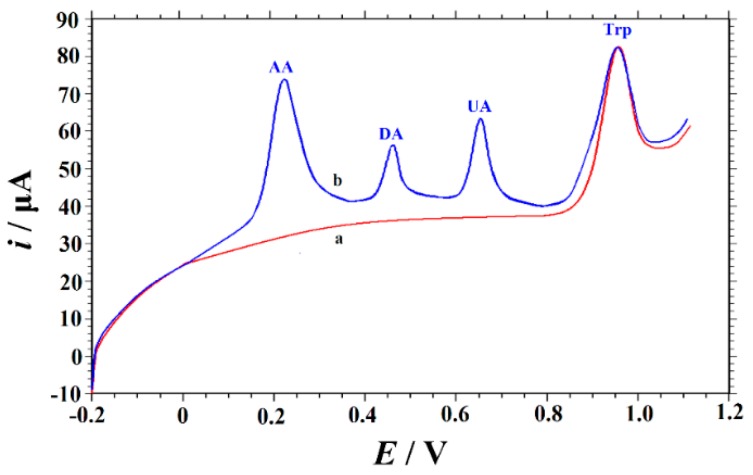
Linear sweep voltammograms of 10 μM Trp in 0.05 M H_2_SO_4_ solution on the Cu_2_O–ERGO/GCE in the absence of (curve a) and presence of 1.0 mM AA, 10 μM dopamine (DA), and 10 μM uric acid (UA) (curve b). Accumulation potential: –0.1 V, accumulation time: 120 s, scan rate: 100 mV s^−1^.

In this part, the effects of some interfering substances that may present in actual samples on the electrochemical oxidation of Trp were examined. Under the optimal conditions, linear sweep voltammetry (LSV) was performed in 0.05 M H_2_SO_4_ solution containing 10 μM Trp in the absence and presence of each interfering substance of different concentrations. Ascorbic acid (AA), uric acid (UA), dopamine (DA) usually coexist with Trp in biological fluids. Therefore, the interference effect of AA, UA and DA on the oxidation of Trp was first studied. AA, UA and DA were found to produce oxidation peaks at 235 mV, 468 mV, and 663 mV, separate from the Trp peak (Figure 8). The experimental results showed that the peak current response of Trp did not change significantly in the presence of 100-fold excess of AA, 20-fold excess of UA and DA. In addition, the effects of other different species were also studied. No substantial changes in the peak current of Trp were observed in the presence of 100-fold excess of glucose, tartaric acid, citric acid, sodium chloride, and potassium chloride. Moreover, in biological fluids as well as some pharmaceutical preparations, Trp often coexists with different amino acids. Therefore, we separately studied the interference effects of different amino acids. Since most amino acids are not electroactive, it was found that 100-fold concentrations of glycine, alanine, valine, leucine, isoleucine, phenylalanine, histidine, and glutamic acid had no effect on Trp oxidation (signal change ≤±5.0%). However, the oxidation peak potential of tyrosine is very close to that of Trp. The results show that 2-fold tyrosine has no obvious interference on the determination of Trp.

### 3.7. Calibration Curve and Limit of Detection

**Figure 9:**
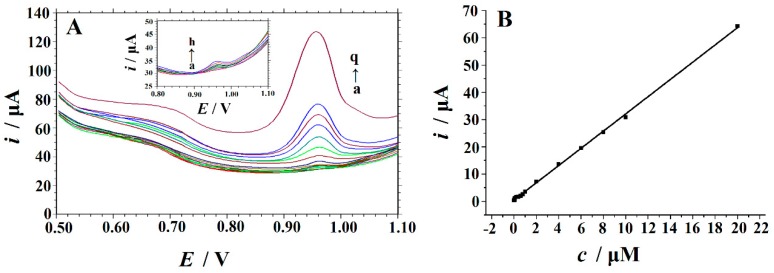
(**A**) SWVs of different concentrations of Trp (a→q: 0.02, 0.04, 0.06, 0.08, 0.1, 0.2, 0.4, 0.6, 0.8, 1.0, 2.0, 4.0, 6.0, 8.0, 10, and 20 μM) on the Cu_2_O–ERGO/GCE in 0.05 M H_2_SO_4_ solution at a scan rate of 100 mV s^−1^. Inset: magnified image of the curve a→h. (**B**) The linear relationship between the *i*_p_ and Trp concentration in the range of 0.02–20 μM. Accumulation potential: –0.1 V; accumulation time: 120 s.

Under the optimized conditions, Trp was quantitatively analyzed by square wave voltammetry (SWV), and the corresponding voltammograms of different concentrations of Trp in the range of 0.02 µM to 20 µM are shown in Figure 9A. The oxidation peak current increased linearly with Trp concentration. The calibration curve of Trp measurement (Figure 9B) showed a linear relationship with Trp concentration (0.02–20 µM). The regression equation is *i*_p_ (µA) = 3.1596c (µM) + 0.4811 (*R*^2^ = 0.9992). The low detection limit can be calculated by using Equation (1):

LOD = 3s_b_/S. (1)

In the formula, s_b_ is the standard deviation of seven blank measurements and S is the sensitivity. The low detection limit was identified at 0.01 µM (*S/N* = 3). The linear range and detection limit of Cu_2_O–ERGO/GCE were compared with other electrodes. As indicated in Table 1, the linear range obtained on Cu_2_O–ERGO/GCE was comparable to and even wider than that of other modified electrodes, and the detection limit was lower than that of most electrodes except PSA/GCE [12] and Pd–Cu@Cu_2_O/N-RGO/GCE [20]. Although a better detection limit (1.9 nM) and a wider linear range (0.01–40 μM) have been achieved on Pd–Cu@Cu_2_O/N-RGO/GCE, the noble metal Pd was used for electrode preparation. In addition, hydrothermal synthesis and chemical reduction were used in the preparation of the electrode. Due to the high cost of Pd and the complex and time-consuming preparation process, its practical application in commercial uses is greatly restricted. These results show that Cu_2_O–ERGO/GCE is a suitable platform for Trp determination. In addition, compared with other carbon-based materials, Cu_2_O–ERGO/GCE has the advantages of low production costs and a simple preparation method.

### 3.8. Repeatability, Reproducibility, and Stability of Cu_2_O–ERGO/GCE

According to some reports [13,14,15], Trp is easily adsorbed onto the electrode surface and fouls the electrodes. In the present work, the most attractive feature of measuring Trp using Cu_2_O–ERGO/GCE is that the electrode surface is easily renewed for the next use. After each measurement, the used electrode was rinsed in 0.1 M H_2_SO_4_ solution and several voltammetric scans in the potential range of 0.5–1.2 V were carried out. The relative standard deviation (RSD) of seven measurements on the same electrode in a 10 μM Trp solution was 1.64%, which indicated the weak adsorption of Trp oxidation product on the electrode surface. In order to demonstrate the stability of Cu_2_O–ERGO/GCE, 100 CVs were performed using 10 μM Trp. It was found that the peak current decreased only 3.47% even after 100 cycles. We also demonstrated the reproducibility by measuring the CV changes in a 10 μM Trp solution obtained on seven different electrodes, and the RSD was calculated to be 4.61%. Furthermore, Cu_2_O–ERGO/GCE long-term stability was also tested. The experimental results showed that the current response of the modified electrode was 94.28% after two weeks in the refrigerator, which indicates that the modified electrode has high stability.

### 3.9. Practical Applications

To explore the application feasibility of Cu_2_O–ERGO/GCE in the determination of Trp, human serum samples and commercial compound amino acid injections were used as real samples. Before analysis, serum samples were prepared according to our previous report [52], then the compound amino acid injection was diluted 100 times with double-distilled water. Thereafter, an aliquot of the sample solutions was diluted with 0.05 M H_2_SO_4_. In order to prevent the matrix effect, a standard addition method was used. The results are shown in Table 3. The recoveries of human serum samples and amino acid injection samples were 97.0–102.3% and 98.0–101.2%, respectively. The results indicated that the electrode had high selectivity and accuracy for the determination of Trp in drugs and biological samples.

## 4. Conclusions

A highly sensitive electrochemical method was established for the determination of Trp by Cu_2_O–ERGO/GCE. On Cu_2_O–ERGO/GCE, Trp undergoes a sensitive oxidation process and a sharp peak is observed. The oxidation enhancement of Trp is due to the synergistic effect of Cu_2_O and ERGO on the surface of the electrode. The results show that the modified electrode has wide linear range, low detection limit, high stability, and good selectivity for the determination of Trp. In addition, the modified electrode has the advantages of good reproducibility and simple preparation, especially its anti-fouling performance to Trp and its oxidation product. The method has been applied to the determination of Trp in human serum samples and commercial amino acid injections. The results indicate that the method can be applied for the determination of Trp concentration in real samples.

## Figures and Tables

**Table 1:** Comparison of results of Trp sensing between this study and previous reports.

Technique	Modified Electrode	Linear Range (μM)	Detection Limit (μM)	Repeatability (RSD%)	Reproducibility (RSD%)	Stability	Reference
^a^ DPV	^d^ EGPU-tAuNP	0.6–2.0	0.053	-	-	-	[10]
DPV	^e^ β-CD/MWCNTs/GCE	1.5–30.5	0.07	6.0	-	20 days	[11]
DPV	^f^ ETPG	0.5–50.0	0.05	-	3.2	a month	[12]
	^g^ PSA/GCE	0.05–10	0.0068	2.7	-	four weeks	[13]
DPV	^h^ rGO/SnO_2_/GCE	1–100	0.04	-	2.87	two weeks	[14]
DPV	^i^ Au-NPs/GCE	0.09–50	0.08	2.4	-	a week	[15]
^b^ SDLSV	^j^ Graphene/ABPE	0.1–10; 10–100	0.06	2.7	4.6	two weeks	[16]
amperometry	^k^ CNF/CPE	0.1–119	0.1	1.0	2.2	an hour	[17]
DPV	^l^ BuCh/GCE	2–60	0.6	-	-	a month	[18]
DPV	^m^ MWNTs/GCE	0.25–100	0.027	2.6	-	two weeks	[19]
DPV	^n^ Pd-Cu@Cu_2_O/N-RGO/GCE	0.01–40	0.0019	2.11	3.78	a month	[20]
^c^ SWV	Cu_2_O–ERGO/GCE	0.02–20	0.01	1.64	4.61	two weeks	This work

^a^ Differential pulse voltammetry; ^b^ Second-order derivative linear sweep voltammetry; ^c^ Square wave voltammetry; ^d^ Gold nanoparticles modified graphite polyurethane composite electrode; ^e^ β-cyclodextrin incorporated with multi-walled carbon nanotubes modified glassy carbon electrode; ^f^ Electrochemically treated pencil graphite electrode (ETPG); ^g^ Poly(sulfosalicylic acid) modified glassy carbon electrode; ^h^ Reduced graphene oxide decorated with tin oxide nanoparticles modified glassy carbon electrode; ^i^ Au nanoparticles modified glassy carbon electrode; ^j^ Graphene modified acetylene black paste electrode; ^k^ Electrospun carbon nanofibers modified carbon paste electrode; ^l^ butyrylcholine modified glassy carbon electrode; ^m^ multi-walled carbon nanotubes modified glassy carbon electrode; ^n^ Pd−Cu@Cu_2_O cubes decorated N-doped reduced graphene oxide modified glassy carbon electrode.

**Table 2:** Detailed data of cyclic voltammograms of 10 µM Trp at different electrodes.

Electrode	*i*_p/_μA	*E*_p/_V
GCE	0.3190	0.994
GO/GCE	4.295	0.995
Cu_2_O–GO/GCE	6.985	0.975
ERGO/GCE	11.48	0.924
Cu_2_O–ERGO/GCE	38.17	0.923

**Table 3:** Determination of Trp in real samples (*n* = 4).

Samples	Labeled/µM	^a^ Detected/µM	Added/µM	^a^ Total Detected/µM	Recovery/%
17AA-I	2.12	2.14 (±0.06)	2.0	4.10 (±0.10)	98.0
18AA-I	4.90	4.97 (±0.13)	5.0	10.03 (±0.29)	101.2
Serum 1	-	1.29 (±0.03)	1.0	2.26 (±0.06)	97.0
Serum 2	-	2.74 (±0.07)	1.0	5.81 (±0.15)	102.3

^a^ Average ±confidence interval, the confidence level is 95%.
